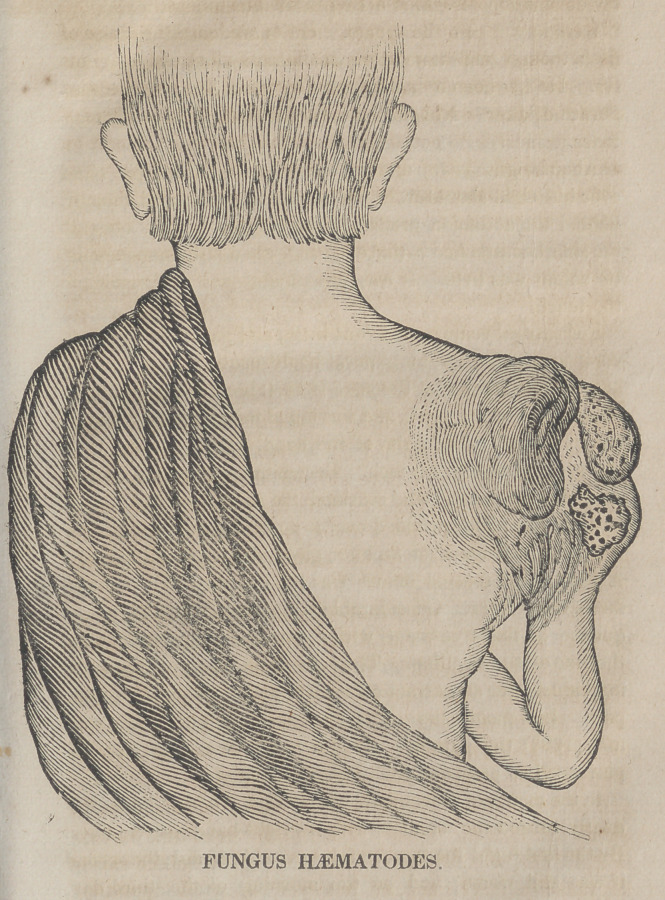# Diagnostic Notices of a Case of Fungus Hæmatodes; Designed as a Contribution to the History of That Disease

**Published:** 1829

**Authors:** 

**Affiliations:** Cincinnati


Art. IV.—*Diagnostic notices of a case of Fungus Hæmatodes; designed as a contribution to the history of that disease.* By the Editor.

The unfortunate subject of the following case, on the 20th of this month, made a visit to Cincinnati, for the purpose of becoming my patient. During his stay of a week, he was seen and examined by a number of my medical friends, most of whom have advised the publication of the history of his disease, together with the annexed drawing of the tumour made by one of them, Dr. Warren. From the rare occurrence of this terrible malady, I have thought proper to follow their advice.

Whether Fungus Hæmatodes is more frequent in one country than another, or is connected with any particular condition of society, I am unable to say. In the Western States it is so uncommon, that most physicians have not seen a single case. Before that now under consideration, I had never, in nearly 30 years, met with but one case. It was in a young woman of the country, and existed on the upper and inner part of one of the lower extremities, with attachments to the pubic and ischiatic bones. The tumour was large and conoidal. She was advised to have nothing attempted except for palliation; her mind ran, however, upon an operation; and she found three physicians willing to undertake it. Uniting in the enterprise, they wielded the scalpel by turns, and continued their slicing until she expired. But let us resume the history of the other case.

FUNGUS HÆMATODES.

Mr. John Anderson, of Kenhawa county, Virginia, aged 46 years, is the person whose disease, I am about to describe. By occupation Mr. A. is a carpenter, but has not worked at his trade for 10 or 12 years. He is now much reduced in flesh, though some time since was athletic, and weighed 220 lbs. He has been a hearty eater of meat, but not an intemperate drinker. Nône of his family ever had Fungus Hæmatodes.

About eight years ago, by accidentally putting his hand over his right shoulder, he discovered a small hard tumour, behind the acromion process of the scapula. It was beneath the skin, but attached to that covering, and nearly immoveable. Its colour was livid. It was not painful, nor even tender to the touch.

For several years it grew but little, and gave him no inconvenience or concern, his general health continuing good. At length, in the spring of the year 1828, it became affected with occasional darting pains, and was found to have attained, at its base, the size of the palm of the hand, with a thickness or elevation of nearly an inch. Becoming alarmed, he applied to an 'Indian doctor' who scarified the surface of the tumour with a razor, and sprinkled over it a powder called 'wet fire,' which, in the course of an hour gave him excruciating pain. A poultice of roasted onions was then applied, and renewed for several days. Considerable sloughing was the consequence of this treatment, with a corresponding reduction in the size of the swelling. The next application by the same individual, was an extract of several herbs, which gave some pain. In a month the ulcer cicatrized. During the summer (1828) the patient's general health was good; he experienced no pain, and the tumour did not grow.

In the month of November, while digging a well, he imprudently turned the windlass with his right hand for two days. By the first night the tumour felt sore and painful; the second it was still worse; and on the morning of the third day, it felt so bad that he desisted. From this time forward it grew rapidly and was much more painful.

Through the ensuing winter it constantly grew worse; paining him both day and night; but more at night, and most when in bed; so that he could not, on an average, lie down more than one third of the usual time. Exposure to cold likewise increased the pain, while heat gave great relief; so that he was induced to rise every night and expose it for hours together, to the action of a hot fire. Even when subjected to the air, in the month of March, during our examinations of it, the pain would increase, and could only be relieved by drawing close to the fire, Throughout this period, however, his general health did not fail in any material degree; he had, according to his own account, no fever; his appetite was good; and his bowels regular, and yet he lost considerable flesh.

Early in February, he again had recourse to the 'Indian doctor,' who directed mercurial ointment, and a poultice of mush (Indian corn meal and boiling water) with some kind of powdered black seeds, which was continued for several weeks, and seemed to mitigate the pain. About the middle of the month, however, a fungus shot out near the lower part of the tumour and continued to grow. In this state of things, he inadvertantly pulled himself up in bed, with his right hand, which hurt him very much, and the next morning the fungus discharged about half a pint of blood; from which time forward, the tumour was affected with greater pain and soreness; and grew more rapidly. The pain was generally dull, but occasionally acute and darting, and it sometimes extended down the humerus. Meanwhile he suffered a rapid emaciation, and about the end of February his feet and legs began to swell.

When he visited Cincinnati, the tumour was of such dimensions, that it required a string twelve inches long to pass over it, from one side of the base to the other; but it was deeply cleft through the centre into two lobes or tuberous masses, the larger lying towards the spine; and between them was a considerable area of ulcerated surface, covered with offensive pus, and a fungus of the size of a hen's egg.—The most projecting of the rounded summits, was about three inches high. In some parts the surface was covered with hard, semi transparent tubercles, of various sizes, which discharged no fluid when they were punctured. Its colour was not unlike that of a ripe mulberry. Most parts of the tumour had a spongy feel, but towards the processus acromion, it was of a cartilaginous hardness. On every side the morbid structure graduated into the sound. The induration and discolouration extended downwards and forwards, quite into the axilla, the glands of which were evidently involved in the diseased structure.

His complexion was sallow; countenance anxious; tongue not particularly foul; pulse habitually frequent; abdomen as well as his feet and legs swollen; respiration difficult, especially in ascending an eminence; and the affected side œdematous. Under percussion this side every where, except over its highest parts, emitted a dull and inelastic sound; and, on resorting to the stethoscope, the respiratory murmur was found to be entirely absent below a horizontal line passing through the upper part of the axilla. The left side sounded well, and the respiratory crepitus was audible throughout. He could only lie on his back. The arm of the affected side was much reduced in size.

The back part of the left shoulder, and the left leg, had a small, subcutaneous tumour, adhering to the skin, which had assumed a livid colour. They were discovered three years before, and appeared to be of the same kind with that on his right shoulder, when it first attracted his attention.—It gave him no pain to press upon or handle them. Had not the first of the group developed itself with the characters of fungus hæmatodes, they might be regarded as cancerous.

In referring to the history of this case we find, first, that the disease occurred in a healthy subject without any obvious cause; secondly, that the tumour cicatrized after the integuments had been ulcerated by escharotics; thirdly, that muscular exertion of the arm and shoulder of the affected side, had a manifest influence, in accelerating the growth and disordering the sensibilities of the part; fourthly, that cold was prejudicial, and external heat palliative.

What were the resources of the profession in this case? The extent of the tumour; the degeneracy of the integuments; the morbid state of the parts in the axilla; the simultaneous existence of other small tumours of the same kind; and the state of the patient's health, in the opinion of all the gentlemen who saw him, rendered a resort to the knife hopeless. To promote absorption from the chest, abdomen and lower extremities, he was advised to use the pil. hydrargyri, and the compound powder of jalap. But his anxieties called for something more; and I was induced to recommend an ointment of the hydriodate of potash, around and upon the base of the swelling, with the internal use of the tincture of iodine by day, and laudanum at night. Others might have preferred to do nothing. But has not iodine set aside both the strumous and the goitrous action, and why might it not supersede, the morbid action in fungus hæmatodes? Who could have *foretold* that scrophula and goitre would disappear before the influence of iodine, or syphilis yield to the action of mercury? In each of these maladies, there is a specific morbid action, with which the effects of their respective remedies, are incompatible, and why should we decree, that the morbid action in fungus hæmatodes can be superseded by no agent in nature? If any one mode of diseased existence, exhibiting specific symptoms, can be cured, by substituting the action of a particular agent, why may not other modes be superseded by other agents? He who does not see a relation of *incompatibility* between syphilis and mercury; small pox and the cow pock; and intermitting fever and arsenic or the sulphate of quina, seems to me to look at the subject with prejudice.

It signifies nothing in the argument, that we employ other means at the same time—means calculated to reduce or exalt the powers of the system, or to correct certain functions, sympathetically disturbed,—unless it can be shown, that *these* means are adequate to the cure. It would be absurd to administer the sulphate of quina in a case of intermittent, attended with a *high* phlogistic diatheses. That condition must first be *reduced*, by blood letting or other sedatives, but all the world knows, that this would not be the *cure* of the malady. That is effected by the Bark, or arsenic, or some other irritant, which substitutes its own irritation for the febrile. This principle of substitution, is more operative in the practice of medicine than many suppose. There are even few acute diseases, which yield to mere depletion; they generally require a superseding agent of some kind, to arrest their progress.

Of course in prescribing iodine in a case of fungus hæmatodes, it should not be done with any definite expectation of a cure. Such an event is only possible: It may come from that medicine, or from some other, or perhaps from none. Because some diseases can be cured, by the operation of certain irritants, it by no means follows, that there are remedies of that class for all. But still, we know not in this respect the limits of our *therapeutia*, and can only reach them by experimental efforts

*Cincinnati, March*, 1829.

## Figures and Tables

**Figure f1:**